# 非小细胞肺癌根治性切除术后复发转移模式研究进展

**DOI:** 10.3779/j.issn.1009-3419.2021.102.50

**Published:** 2022-01-20

**Authors:** 显平 刘, 晓 李, 帆 杨

**Affiliations:** 100044 北京，北京大学人民医院胸外科 Department of Thoracic Surgery, Peking University People's Hospital, Beijing 100044, China

**Keywords:** 肺肿瘤, 根治性切除术, 复发转移, 动态模式, Lung neoplasms, Radical resection, Recurrence and metastasis, Dynamic pattern

## Abstract

非小细胞肺癌根治性切除术后复发转移率仍然很高，其复发转移的危险因素得到了广泛研究，但术后复发风险随时间变化的动态模式研究相对欠缺。复发风险的动态模式能够直观反映风险人群在任何时间点的复发率。在这篇综述中，通过回顾既往文献，总结了非小细胞肺癌根治性切除术后动态复发转移模式的特点以及影响复发转移模式的临床因素，以期筛选出高复发风险的特定人群并给予个性化的随访策略及诊治。

## Abstract (en)

The rate of recurrence and metastasis of non-small cell lung cancer after radical resection is still very high. The risk factors for recurrence and metastasis have been extensively studied, but the dynamic pattern of postoperative recurrence hazard over time is relatively lacking. The dynamic recurrence hazard rate curve is applied to describe the rate of recurrence at any point time among the "at-risk" patients. In this article, by reviewing the previous literature, the characteristics of the dynamic recurrence and metastasis pattern after radical resection of non-small cell lung cancer and the clinical factors affecting the recurrence and metastasis pattern are summarized, in order to screen out specific populations with high recurrence risk and give them personalized follow-up strategy and diagnosis and treatment.

肺癌是中国乃至全世界第一大恶性肿瘤，发病率及病死率位于所有恶性肿瘤前列，非小细胞肺癌（non-small cell lung cancer, NSCLC）约占所有肺癌的85%-90%^[1]^。在中国，2018年肺癌发病人数77万，占全球发病人数的37%，2018年中国肺癌死亡人数69万，占全球死亡人数的39%^[2]^。发现肺癌时，诊断为早期（Ⅰ期-Ⅱ期）的患者占16%，局部中晚期（Ⅲ期）患者占22%，晚期患者占57%^[1]^。对于Ⅰ期、Ⅱ期和部分Ⅲa期NSCLC患者，根治性手术切除是最佳治疗选择，公认的标准术式为“肺叶切除+系统性纵隔淋巴结清扫”，但仍有约30%-55%的患者根治性切除后会复发转移导致治疗失败^[3]^。根治性切除后的NSCLC患者根据术后病理分期的不同，5年无复发生存率差异很大，Ⅰb期-Ⅲb期患者的5年无病生存率（disease-free survival, DFS）在17%-73%之间，有待进一步提高^[4]^。除此之外，随着分期的提高，复发的部位也不一样，分期为Ⅰ期时，局部复发率和远处复发率相近，约为10%；分期为Ⅱb期-Ⅲa期时，局部复发率为12%-15%，而远处复发率则达到40%-60%^[3, 5-7]^。

胸外科医生对NSCLC根治性切除后复发转移的情况十分关注，希望能够探索清楚其中的原理和规律，以帮助后续的进一步治疗。国内外的很多指南^[8-10]^已经发布了很多关于NSCLC根治性切除术后的随访策略，其很大的一部分内容就是建立在肺癌术后的复发转移模式上。因此进一步深入了解NSCLC根治性切除术后的复发转移模式十分重要。

尽管已有不少文献^[7, 11-15]^报道了NSCLC根治性切除术后的复发转移情况，但其中绝大多数研究的是肺癌根治性切除术后复发转移的独立危险因素及保护因素，而复发转移模式还应充分探索并研究复发风险随时间变化的规律，以期在术后的具体时间段内给予更加精准的监测。本文检索了2000年1月-2021年3月期间发表的相关文献，将NSCLC根治性切除术后的复发转移模式进行了汇总，以期寻找出复发转移模式的特点从而为手术切除后的随访策略提供参考。

## 1 根治性切除术后复发的机制

国内外已有大量研究报道肺癌根治性切除术后复发的机制，这些研究表明根治性切除需从宏观和微观两方面保证其切除的完整性。术后复发的机制可用肿瘤休眠假说^[16-18]^来解释，该种假说设定所有的肺癌患者体内均存在隐匿型微转移，且处于一种相对稳定的状态，大部分不会促进肿瘤生长，但是手术会破坏这种稳定状态，从而刺激休眠的肿瘤细胞增殖，从而加速复发过程，最终导致复发。此外，有人提出患者体内通常存在隐匿的癌细胞，如循环肿瘤细胞或播散性肿瘤细胞，这些癌细胞是目前标准分期办法（如现代影像学检查）无法检测到的^[19-22]^，说明目前我们有可能低估了真正的肿瘤分期。同时，手术过程中对肿瘤本身的处理也有可能导致癌细胞的扩散^[19, 23]^。有研究^[22, 24-26]^发现肺癌切除过程中循环肿瘤细胞或播散性肿瘤细胞的存在与患者预后存在相关性，循环肿瘤细胞在肺癌诊断和治疗中也有一定程度的应用^[27, 28]^，目前仍有许多研究者在探索循环肿瘤细胞的临床意义，仍无一致性共识。

肺癌干细胞在肺癌转移中也有不可忽视的作用，它是肺癌转移的启动者和执行者。肺癌干细胞的细胞标志物表达如CD133通过上调基质金属蛋白酶9和缺氧诱导因子-1α促进肺癌干细胞转移；还有证据表明，肺癌干细胞与上皮-间质转化密切相关，从而促进肺癌转移^[29]^；癌症干细胞和病灶内炎性微环境的相互作用促进其转移^[30]^。

近年来有研究^[31, 32]^表明肿瘤相关巨噬细胞（tumor associated macrophages, TAM）及肿瘤免疫微环境（tumor microenvironment, TME）与肿瘤迁移、侵袭和转移密切相关，TAM是TME的主要成分，在转移中起着至关重要的作用。TAM主要通过分泌基质金属蛋白酶、丝氨酸蛋白酶和组织蛋白酶来促进肿瘤细胞的侵袭和迁移，这些蛋白酶可以修饰细胞-细胞连接并破坏基底膜^[33]^。有研究^[34, 35]^称，TME本身也促进肺癌向远处转移。除此之外，TME中还存在肿瘤相关中性粒细胞（tumor-associated neutrophil, TAN），与非消退性炎症关系密切^[36]^。TAN通过释放各种趋化因子，在炎症部位激活和募集单核细胞/巨噬细胞^[37]^，并且募集的TAM会反过来影响TAN的功能，它们在维持TME及促进肿瘤进展中起到很重要的作用^[36]^。

关于肺癌根治性切除术后复发转移的机制目前没有一致的共识，仍然有很多研究在探索其中的奥秘。

## 2 复发转移的危险因素

肿瘤原发灶-淋巴结-转移（tumor, node and metastasis, TNM）分期仍然是目前公认影响肺癌根治术后复发转移最强有力的因素^[38, 39]^，并且随着该分期的不断更新及完善，其有效性及区分度也更加明显。但是，即使是同一分期的患者，在根治性切除术后复发转移也存在很大的差异，这提示仅仅依靠TNM分期是不足的。对于那些可根治性切除及可能从辅助治疗中获益的患者人群，准确预测可能的复发转移有助于指导后续辅助治疗的实施，提高其生存获益。

目前得到大多数认可的复发转移因素包括高龄、高癌胚抗原（carcinoembryonic antigen, CEA）水平、吸烟史、气腔内播散、胸膜浸润、脉管内浸润、淋巴结转移数目、正电子发射计算机断层显像（positron emission tomography-computed tomography, PET-CT）的标准吸收值的最大值（standardized uptake value max, SUVmax）^[7, 14, 40-45]^。自2011年提出新的腺癌分类以来，很多研究^[12, 46-49]^发现实性及微乳头为主型的病理亚型是复发转移的高危因素，有的研究^[11, 50]^还发现含有实性或微乳头成分（比例 > 5%）的肺癌人群也预示更差的无复发生存率。随着胸部薄层电子计算机断层扫描（computed tomography, CT）的普及，很多磨玻璃结节及实性小结节被发现，其中一部分经病理确诊为早期肺癌，在这些人群中，纯实性结节预后不良，而含有磨玻璃成分是有利的预后因素^[51-53]^。在磨玻璃结节肺癌人群中，表现为纯磨玻璃结节的人群经手术切除后几乎没有复发，而表现为亚实性结节的人群较纯磨玻璃结节人群更易复发，在表现为亚实性结节人群中，高实性占比、高实性成分比例（consolidation tumor ratio, CTR）值也与术后复发转移显著相关^[54, 55]^。肺癌术后复发转移受很多因素影响，目前关于这方面的研究也多为回顾性研究，需要使用临床相关模型进行更大规模的研究，从而进一步扩展其在临床中的实用性。

## 3 复发转移的模式（时空特征）

### 3.1 复发转移模式的空间特征

近年以来，NSCLC根治性切除术后复发转移的空间特征得到越来越多的关注。根据既往文献及胸外科领域的一致共识，手术切缘、吻合口、支气管残端、同侧胸壁、同侧胸膜、同侧肺或同侧区域淋巴结复发定义为局部复发，对侧肺及淋巴结（包括颈部或腹部淋巴结）、脑、骨、肝等实体器官复发定义为远处转移。远处转移是肺癌复发最常见的部位，局部复发所占比例较小，多项研究^[7, 56-58]^的结果均支持这一结论。表 1展示了NSCLC根治性切除术后局部复发率与远处转移率的对比。既往文献^[57, 59-61]^结果显示，早中期NSCLC患者接受根治性切除手术后，远处转移的比例为14%-25.5%，局部复发比例为3.2%-8.7%（具体结果可见表 1）。中位随访时间57个月，2000年-2018年我中心共2, 751例有完整随访信息的肺腺癌患者接受根治性切除后17.0%（469/2, 751）出现了复发转移，其中11.7%的患者首次复发部位为远处转移，仅有4.5%的患者首次复发部位为局部复发。

**表 1:** NSCLC术后复发分布 The recurrence distribution of post-operative NSCLC

Author	Year	Total(*n*)	TNM stage	Median follow-up (mon)	Overall recurrence rate	Local recurrence rate	Distant metastasis rate
Nakagawa^[59]^	2008	397	Ⅰ	74.2	21.9%	7.6%	14.4%
Koo^[61]^	2011	310	Ⅰ-Ⅱ	60.0	34.2%	8.7%	25.5%
Demicheli^[64]^	2012	1, 506	Ⅰ-Ⅲa	60.0	25.8%	6.8%	19.0%
Hung^[79]^	2012	756	Ⅰ	67.7	28.2%	6.7%	21.4%
Lou^[60]^	2013	1, 294	Ⅰ-Ⅱ	35.0	19.9%	5.2%	14.7%
Zhu^[57]^	2014	994	T1a-2bN0M0	73.2	25.7%	3.2%	22.5%
Yamauchi^[56]^	2015	1, 374	Ⅰ-Ⅲa	-	36.5%	9.7%	26.8%
Wong^[80]^	2016	9, 001	Ⅰ-Ⅲ	60.0	33.8%	12.3%	21.5%
Watanabe^[65]^	2016	829	Ⅰ-Ⅲb	65.6	33.1%	15.4%	17.6%
Zhang^[81]^	2018	2, 017	Ⅰ-Ⅲ	36.2	31.8%	-	-
Watanabe^[66]^	2020	1, 289	Ⅰ-Ⅲa	47.4	11.8%	-	-
NSCLC: non-small cell lung cancer; TNM: tumor, node and metastasis.							

### 3.2 复发转移模式的时间特征

复发转移风险随时间变化的动态曲线可以直观地表述其时间特征，通常使用平滑风险函数法作为工具来绘制，这种方法可描述“有风险”患者在任何时间点的复发率^[62, 63]^。Zhu等^[57]^的研究纳入了994例接受根治性切除的早期（T1a-2bN0M0）NSCLC患者，发现复发曲线呈“双峰”型，即术后1.6年达到第一个复发高峰，随后复发风险逐渐降低，到术后8.8年时达到第二个复发高峰，除此之外，在其亚组分析中部分亚组也发现了相似的“双峰”复发模式。Yamauchi等^[56]^回顾了2003年-2009年海德堡大学附属医院收治的1, 374例接受根治性切除+系统淋巴结清扫NSCLC患者的复发转移情况，其风险曲线也呈“双峰”型，术后10个月达到第一个复发高峰，术后第8年出现第二个复发高峰，亚组分析中局部复发与远处转移的风险模式相似，但远处转移的风险在整个随访期间均高于局部复发。从已发表的国内外研究来看，接受根治性切除的NSCLC患者总体复发曲线基本都呈典型的“双峰”型，即术后的较短时间内（通常2年内）达到首个复发高峰，第二个复发高峰出现在术后较长时间段内（> 5年），这种复发模式可用上一部分的肿瘤休眠假说来解释。

### 3.3 复发转移模式与性别的关系

NSCLC复发转移模式可能与性别相关。Demicheli等^[64]^回顾性分析了1, 506例NSCLC患者数据，发现性别可能影响复发转移模式，研究中显示男性及女性均在术后7个月-9个月出现第一个复发高峰，而第二个复发高峰出现的时间男性（约18个月-20个月）比女性（约24个月-26个月）早约6个月。除此之外，Watanabe等^[65]^纳入了日本横滨胸外科联盟所属9家医院接受根治性切除的829例NSCLC患者，研究发现男性中最高的复发风险出现在术后6个月-8个月，而女性最高复发风险出现在术后22个月-24个月，比男性晚约16个月，同时该研究还发现男性及女性的最高复发风险出现时间不受病理组织类型、年龄及病理分期的影响。复发转移模式因性别不同而存在差异，可能的解释为与血管生成相关的内部环境在男性和女性之间是不同的，不同的内环境可能选择了具有不同特征的肿瘤细胞，也有可能是男性和女性中对它们的作用不同，从而导致微转移休眠期的不同停留时间，从而导致复发风险高峰出现时间的不同^[64]^。

### 3.4 复发转移模式与病理类型的关系

NSCLC的病理类型也会影响复发转移模式，但不同的文献报道有不同的见解。Yamauchi等^[56]^的研究还发现，浸润性腺癌和鳞癌的复发风险曲线相似，均呈较典型的“双峰型”，但在整个随访期间，浸润性腺癌患者的复发风险一直略微高于鳞癌患者，提示不同的病理类型的NSCLC生物学行为不一样，导致了不同的复发风险。但在Watanabe等^[65]^的研究中，鳞癌患者复发风险高于浸润性腺癌患者，鳞癌患者复发风险率在术后第一年呈现为“尖峰”，其次是4个-5个小峰，而浸润性腺癌患者复发风险在术后6个月-14个月逐渐增加，之后复发风险逐渐平缓下降。

Watanabe等^[66]^的另外一篇研究中，纳入1, 289例根治性切除术后的肺浸润性腺癌患者，发现含有微乳头成分的患者术后1年内复发风险曲线出现最初的宽高峰，而无微乳头成分的患者在术后2年左右复发风险曲线出现平缓的高峰，除此之外，微乳头成分组在整个随访期间复发风险均高于无微乳头成分组，结果提示含有微乳头成分的患者术后早期复发的风险较高，且复发风险长期存在，复发高峰相较于无微乳头成分患者提前约1年。有研究^[11]^表明，微乳头成分的存在与广泛的淋巴结微转移和预后不良有关，Kamiya等^[67]^研究还发现微乳头成分中的肿瘤细胞很可能获得了失巢凋亡抗性并促进了不依赖锚定的生长，从而增强了肿瘤细胞在脉管系统和淋巴循环中的增殖能力，最后导致复发提前且复发风险升高。总体来看，含有微乳头成分肺腺癌患者不仅复发风险高，同时复发时间提前，需要值得我们重视并给予个性化的随访策略。

### 3.5 复发转移模式与表皮生长因子受体（epidermal growth factor receptor, *EGFR*）

基因突变及靶向治疗的关系随着分子检测技术的进步，肺癌靶向治疗和驱动基因检测相关研究如火如荼，其中最受瞩目的就是*EGFR*基因突变。多项大型临床试验结果^[68-71]^提示，*EGFR*敏感突变的中晚期肺癌患者，EGFR酪氨酸激酶抑制剂（EGFR-tyrosine kinase inhibitor, EGFR-TKI）一线治疗已成为标准模式，也写进了多个地区和国家的肺癌诊疗指南^[9]^。尽管*EGFR*基因突变状态是EGFR-TKI治疗效果最强的预测因素，但*EGFR*基因突变是否是肺癌预后的预测因素仍存在着很大的争议。晚期NSCLC的患者接受一代EGFR-TKI治疗中，脑转移为最常见的疾病进展模式^[72-74]^。ADJUVANT研究^[75]^分析探索了早中期（Ⅱ期-Ⅲa期）*EGFR*敏感突变肺癌患者术后辅助靶向治疗的复发转移模式，结果证实辅助吉非替尼治疗对于控制术后复发转移的疗效要优于辅助长春瑞滨/顺铂（vinorelbine/cisplatin, VP）化疗，尤其是控制颅外转移方面；但在中枢神经系统转移方面，吉非替尼治疗组中位无病生存期（disease free survival, DFS）并没有显著优于化疗组，同时吉非替尼治疗组脑转移占总体复发的27.4%，辅助化疗组占24.1%，两组结果相近。在复发的时间分布上，化疗后出现脑转移的时间远早于吉非替尼，在术后12个月-15个月达到高峰，而吉非替尼要到36个月才会出现高峰；在颅外转移方面，不管在发生时间上还是发生率上吉非替尼组均低于化疗组，因此无论是脑转移还是颅外转移，辅助吉非替尼治疗组都显著推迟了复发风险的高峰时间。这些研究提示对于*EGFR*突变的患者群体，无论是接受化疗还是EGFR-TKI靶向治疗脑转移控制仍欠佳，且其复发转移的时间特征也十分有趣。Matsumura的团队^[76]^通过多中心倾向匹配分析发现，*EGFR*突变不是肺腺癌术后复发的危险因素。Deng等^[77]^纳入了1, 512例接受基因检测的根治性切除术后肺腺癌患者，发现*EGFR*突变是影像学为实性、以腺泡型/乳头型/浸润性黏液腺癌为主、病理分期为Ⅱ期/Ⅲ期患者的独立不良预后因素，除此之外，*EGFR*突变肺腺癌患者脑和骨转移多于野生型。在排除接受靶向治疗的前提下，*EGFR*基因突变状态是否会影响肺癌术后的复发转移模式仍存在争议，值得我们进一步研究。

## 4 结语

在2021年发表的《非小细胞肺癌术后随访中国胸外科专家共识》^[78]^中，通过检索国内外数据库及现行临床指南（如美国国立综合癌症网络指南、中国临床肿瘤学会指南等），根据患者病理分期、是否接受术后辅助治疗及有无基因突变详细阐述了不同的随访方案。但更加个性化的分类指标，如肺癌病理亚型、性别等，其复发转移模式表现出特殊的时空特征，适当在术后增加随访频率及特殊部位的复查，能够尽早发现可能存在的复发转移部位，有助于后续治疗。

NSCLC根治性切除术后复发转移是肿瘤诊疗过程中遭遇失败的主要原因，其复发转移模式值得我们进一步探索及研究，特别是其时间特征，有助于我们筛选出在特定时间段内易复发的人群并指导后续随访及诊治。但是关于复发转移模式尚无统一的意见，仍需得到进一步验证和探索。
